# Specific inflammatory profile of acute ischemic stroke patients with left atrial enlargement

**DOI:** 10.3389/fcvm.2023.1190857

**Published:** 2023-07-19

**Authors:** Julia Fontaine, Simon Leboube, Thomas Bochaton, Hélène Thibault, Camille Amaz, Tae-Hee Cho, Alexandre Paccalet, Claire Crola Da Silva, Suzanne Duhamel, Marielle Buisson, Lucie Rascle, Gabriel Bidaux, Michel Ovize, Norbert Nighoghossian, Laura Mechtouff

**Affiliations:** ^1^ Stroke Department, Hospices Civils de Lyon, Bron, France; ^2^ Univ-Lyon, CarMeN Laboratory, Inserm U1060/INRA U1397, Université Claude Bernard Lyon 1, Lyon, France; ^3^ Cardiac Intensive Care Unit, Hospices Civils de Lyon, Lyon, France; ^4^ Department of Cardiovascular Functional Exploration, Hospices Civils de Lyon, Lyon, France; ^5^ Clinical Investigation Center-INSERM 1407, Hospices Civils de Lyon, Lyon, France

**Keywords:** inflammatory, left atrial enlargement, ischemic stroke, VCAM-1, SST2

## Abstract

**Background:**

The inflammatory process underlying atrial myopathy may affect the inflammatory response activated in acute ischemic stroke (AIS).

**Objectives:**

We aimed to assess whether left atrial enlargement (LAE) as a marker of atrial myopathy is associated with a different profile of circulating inflammatory markers in AIS patients.

**Methods:**

HIBISCUS-STROKE is a cohort study including anterior circulation AIS patients treated with mechanical thrombectomy following MRI. Ten circulating inflammatory markers were measured at admission and 6, 24, and 48 h after admission. LAE was defined as a left atrial volume index (LAVi) ≥34 ml/m^2^. A multiple logistic regression model was performed to detect an independent association between the area under the curve (AUC) of these markers and LAE.

**Results:**

We included 143 patients. Of them, 85 (59.4%) had LAE. On univariable analysis, we found that patients with LAE had higher soluble form suppression of tumorigenicity 2 (sST2), soluble tumor necrosis factor receptor I (sTNFR1), and vascular cellular adhesion molecule-1 (VCAM-1) AUC, were older, mostly female, had a higher National Institutes of Health Stroke Scale (NIHSS) score and blood glucose level at admission, had more often hypertension, and a cardioembolic source of AIS, such as atrial fibrillation, while they were less frequently current smokers and had a lower rate of tandem occlusion than patients without LAE. On multivariable analysis, we found that among circulating inflammatory markers, only high VCAM-1 (OR: 9.13, 95% CI: 3.21–25.9) and sST2 (OR: 3.40, 95% CI: 1.68–6.86) AUC remained associated with LAE.

**Conclusions:**

High VCAM-1 and sST2 levels within the first 48 h are associated with LAE in AIS patients.

## Highlights

• Left atrial enlargement (LAE) was independently associated with higher vascular cellular adhesion molecule-1 (VCAM-1) and soluble form suppression of tumorigenicity 2 (sST2) within the first 48 h in acute ischemic stroke (AIS) patients.
• VCAM-1 levels were higher at baseline and remain increased within the first 48 h in AIS patients with LAE.
• sST2 levels were higher from 6 h after admission in AIS patients with LAE.

## 1. Introduction

Atrial fibrillation (AF) is the most common cardiac arrhythmia and a major cause of stroke, heart failure, sudden death, and cardiovascular morbidity (1). Studies have found that left atrial enlargement (LAE) as a marker of atrial myopathy is associated with an increased risk of ischemic stroke and all-cause of death despite the controversy surrounding this (2, 3). The association between atrial myopathy and stroke may be unrelated to AF (4, 5). Experimental and clinical data indicate that inflammation is implicated in the pathophysiology of atrial remodeling. Inflammatory markers such as CRP, interleukin (IL)-6, -2, -8, tumor necrosis factor-α (TNF-α), monocyte chemoattractant protein (MCP-1), soluble form suppression of tumorigenicity 2 (sST2), vascular cellular adhesion molecule-1 (VCAM-1), and soluble intercellular adhesion molecule-1 (sICAM-1) have also been associated with AF, AF incidence, and recurrence (6–15). The inflammatory process is also accompanied by ischemia-reperfusion damage related to acute ischemic stroke (AIS) (16, 17). Indeed, ischemic insult elicits a strong neuroinflammatory response orchestrated by proinflammatory cytokines, which may offset the benefit of reperfusion (16, 18).

The question whether the inflammatory substrate underlying atrial myopathy may affect the overall inflammatory response in AIS has not yet been answered.

In the present study, we aimed to assess whether LAE is associated with a different profile of circulating inflammatory markers in AIS patients.

## 2. Methods

### 2.1. Study population

The design and methods of HIBISCUS-STROKE have been published previously (19). Briefly, from the year 2016, patients admitted to the Lyon Stroke Center for an anterior circulation AIS with large vessel occlusion (LVO) treated with mechanical thrombectomy (MT) using brain MRI were included in this cohort. Patients with active disease resulting in systemic inflammation were excluded. We collected peripheral blood samples from each patient at admission before performing any reperfusion therapy and at 6, 24, and 48 h after admission. Baseline data on medical history, risk factors, and demographic characteristics were collected at admission. Board-certified neurologists assessed the neurological status using the National Institutes of Health Stroke Scale (NIHSS) score at admission and the modified Rankin Scale (mRS) at 3 months during a face-to-face follow-up visit. Stroke subtype was classified using the Trial of Org 10172 in Acute Stroke Treatment (TOAST) criteria (20). The study was approved by the local ethics committee, and all subjects or their relatives signed an informed consent form (IRB number: 00009118).

### 2.2. Blood sampling protocol

Sera were prepared and stored at −80°C within a 3-h delay at the NeuroBioTec biobank (CRB-HCL: BB-0033-00046, France). All samples were thawed only once for study measurements. CRP, IL-6, IL-8, and IL-10 were measured using an enzyme-linked immunosorbent assay (ELISA) kit (Affymetrix, eBioscience). Monocyte Chemoattractant Protein-1 (MCP-1), soluble tumor necrosis factor receptor I (sTNFR1), sST2, sP-selectin, matrix metalloproteinase-9 (MMP-9), and VCAM-1 were measured using the R&D systems ELISA kit (R&D Systems, Minneapolis, Minnesota).

### 2.3. Transthoracic echocardiography

Two-dimensional and 3D left atrial volume indices (LAVis) were assessed by transthoracic echocardiography. Left atrial enlargement (LAE) was defined as a LAVi ≥34 ml/m^2^.

### 2.4. Brain imaging

MRIs were conducted using 1.5-T Intera or 3-T Achieva scanners (Philips, Best, the Netherlands). The MRI protocol included fluid-attenuated inversion recovery (FLAIR), time-of-flight MR angiography (TOF-MRA), T2-gradient echo, and diffusion-weighted imaging (DWI) sequences. The DWI lesion was outlined using a semiautomated method (3D Slicer, https://www.slicer.org). The DWI-based Alberta Stroke Program Early CT Score (ASPECTS) was assessed (21). The Thrombolysis in Cerebral Infarction (TICI) score was used to assess angiographic reperfusion, and the treatment was considered successful if the score was 2b, 2c, or 3 (22). Hemorrhagic transformations were evaluated on day-1 CT using the European Co-operative Acute Stroke Study-II (ECASS II) classification (23). The follow-up MRI protocol at day 6 included FLAIR sequence.

### 2.5. Statistical analysis

Continuous variables are expressed as means [standard deviation (SD)] or medians [interquartile range (IQR)] depending on their distributions, and categorical variables are given as percentages. Medians were compared using the Mann–Whitney or Kruskall–Wallis test. Percentages were compared using Fisher's exact test. If data were missing for circulating inflammatory biomarkers, we employed imputation methods using the means between the previous and the next observation. If admission or 48 h data were missing, we performed the median slope to replace missing data. The normality of distributions was assessed graphically and by using the Shapiro–Wilk test. The association between each variable and LAE was measured by calculating the odds ratio (OR) and 95% confidence intervals (CI) using simple logistic regression. A multiple logistic regression model was then performed to assess the relationship between the area under the curve (AUC) of circulating inflammatory biomarkers within the first 48 h and LAE. Statistically significant covariates in univariate analyses (*p* < 0.05) that were supposed to be causal were implemented through a backward stepwise procedure with a removal criterion of *p* > 0.05. The multivariable logistic regression was, therefore, adjusted for age, sex, hypertension, current smoking, NIHSS score, glucose level on admission, atrial fibrillation, and tandem occlusion. Statistical testing used a two-tailed *α* level of 0.05. The data were analyzed using Stata Version 15™ (StataCorp, College Station, Texas 77845 USA) and GraphPad Prism (San Diego, California, USA).

## 3. Results

### 3.1. Study population

**Figure 1:**
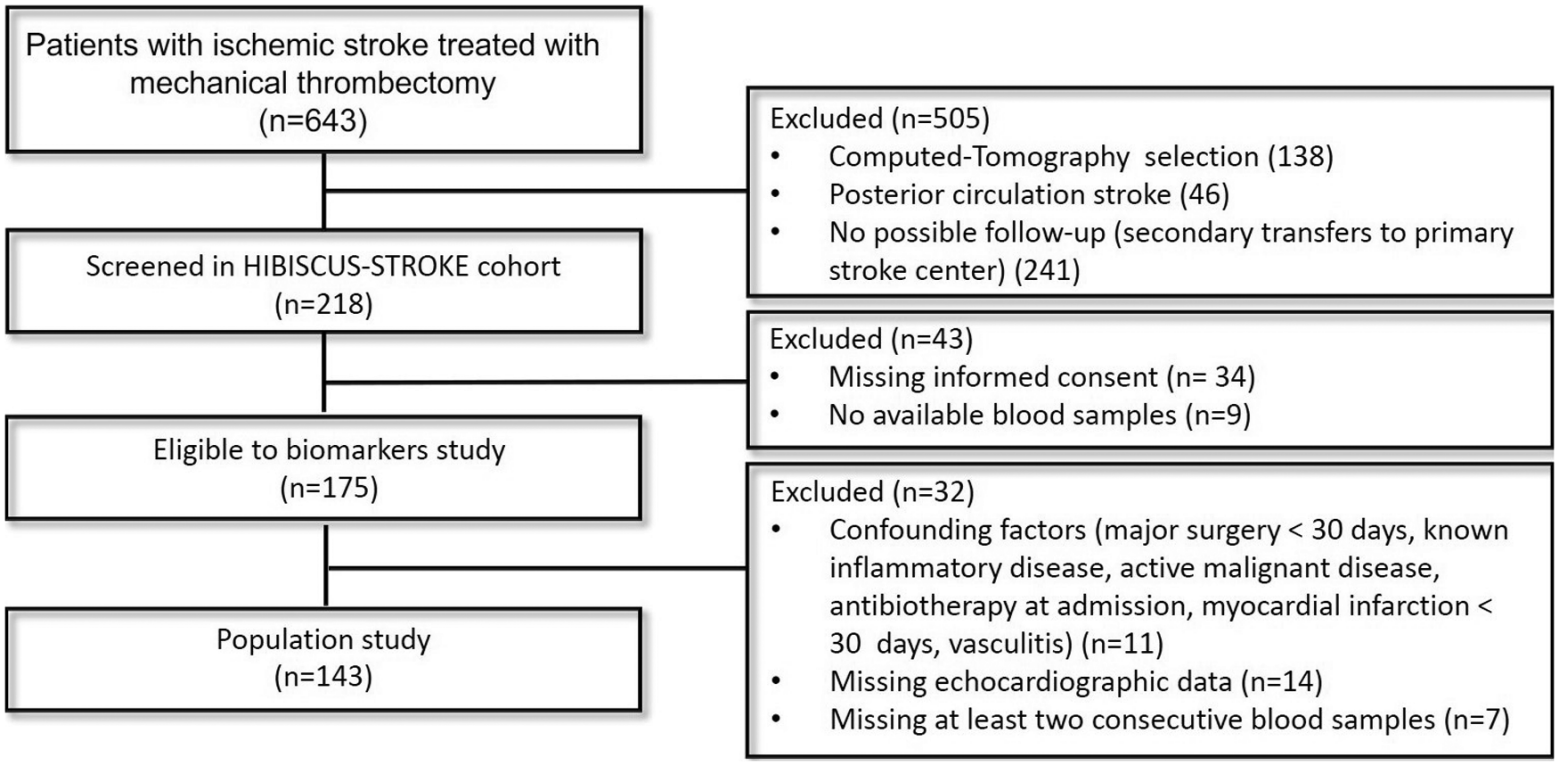
Study flowchart.

Among AIS patients treated with MT in our institution between October 2016 and April 2019, 143 were included (Figure 1). Excluded patients were older (71.6 ± 14.8 years vs. 68.1 ± 15.5 years, *p* = 0.016), were less likely male [234 (46.8%) vs. 88 (61.5%), *p* = 0.002], had a higher baseline National Institutes of Health Stroke Scale (NIHSS) score (16 [11–21] vs. 15 [9–19], *p* = 0.015), and were less likely to have an M1 segment middle cerebral artery [242 (48.4%) vs. 90 (62.9%), *p* = 0.003] or an intracranial internal carotid artery [140 (8.0%) vs. 27 (18.9%), *p* = 0.028] occlusion. The median LAVi was 36.9 [27.1–50.8] ml. Eighty-five (59.4%) patients had LAE. Patient characteristics are detailed in Table 1.

**Table 1:** Main characteristics of study patients according to the presence of left atrial enlargement (LAE).

	No LAE (*n* = 58)	LAE (*n* = 85)	*p*-value
Age, years	60.2 ± 15.4	73.5 ± 13.0	**<0**.**001**
Male	42 (72.4)	46 (54.1)	**0**.**04**
Hypertension	20 (34.5)	48 (56.5)	**0**.**01**
Diabetes	8 (13.8)	15 (17.7)	0.65
Hyperlipidemia	12 (20.7)	24 (28.2)	0.33
Current smoking	20 (34.5)	12 (14.1)	**0**.**01**
Coronary artery disease	8 (13.8)	15 (17.7)	0.65
NIHSS score	13 [8–17]	17 [11–20]	**0**.**03**
Glucose level, mmol/L	5.86 [5.50–6.99]	6.57 [5.72–8.20]	**0**.**01**
Atrial fibrillation	7 (12.1)	56 (65.9)	**<0**.**001**
Etiology			**<0**.**001**
Cardioembolism	10 (17.2)	65 (76.5)	
Large-artery atherosclerosis	15 (25.9)	6 (7.1)	
Other	13 (22.4)	2 (2.4)	
Undetermined	20 (34.5)	12 (14.1)	
Thrombus location			
M1 MCA segment	36 (62.1)	54 (63.5)	0.86
M2 MCA segment	11 (19.0)	13 (15.3)	0.65
ICA terminus	11 (19.0)	16 (18.8)	1.00
Tandem occlusion	18 (31.0)	14 (16.5)	**0**.**04**
ASPECTS	7 [6–8]	7 [6–9]	0.10
Intravenous thrombolysis	33 (56.9)	41 (48.2)	0.39
Successful reperfusion	43 (74.1)	73 (85.9)	0.09
Stroke onset to groin puncture, min^c^	216 [147–395]	225 [165–373]	0.91
PH type 1 or 2 or SAH	2 (3.5)	5 (5.9)	0.70
LAVi, ml/m^2^	26.0 ± 4.8	51.4 ± 16.3	**<0**.**001**
LVEF, %^d^	60.9 ± 5.5	56.5 ± 13.3	0.19
LVEDVi, ml/m^2^^e^	49.3 ± 11.9	54.2 ± 26.7	0.99
IVS thickness, mm^c^	10.2 ± 1.8	10.7 ± 2.5	0.27

NIHSS, National Institute of Health Stroke Scale; MCA, middle-cerebral-artery; ICA, internal carotid artery; ASPECTS, Alberta Stroke Program Early CT Score; PH, parenchymal hematoma; SAH, subarachnoid hemorrhage; LAVi, left atrial volume index; LVEF, left ventricular ejection fraction; LVEDVi, left ventricular end-diastolic volume index; IVS, interventricular septum. Variables are displayed as absolute number, mean ± SD, or median (25th–75th percentiles) as appropriate.

The bold values indicates a *p* value  < 0.05.

^a^ Seventeen patients with missing data.

^b^ Thirty-five patients with missing data.

^c^ Fourteen patients with missing data.

^d^ Three with missing data.

^e^ Forty-seven with missing data.

### 3.2. Factors associated with LAE

**Figure 2:**
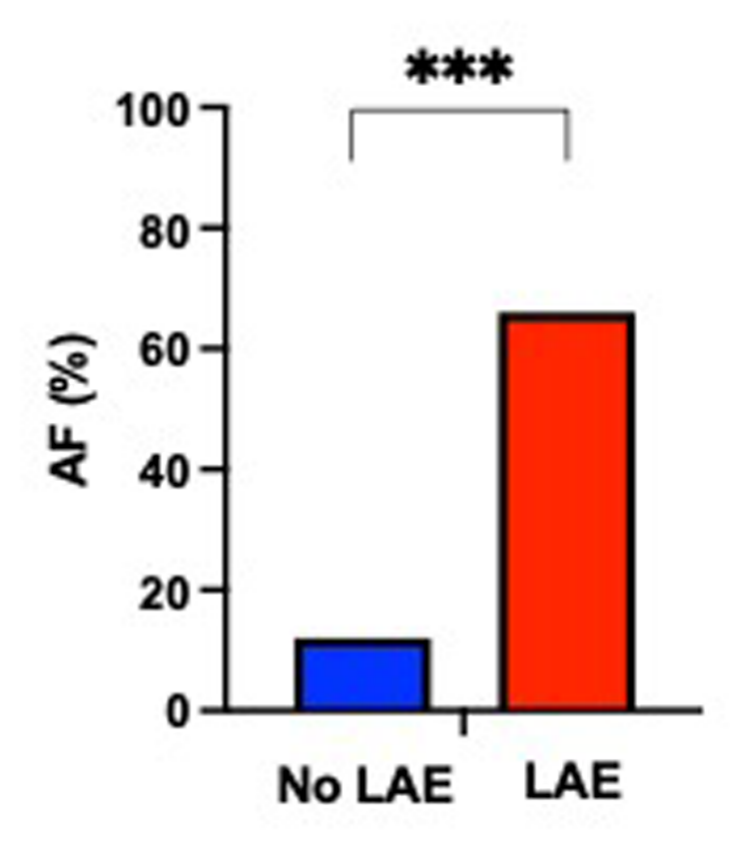
Proportion of patients with atrial fibrillation (AF) according to the presence of left atrial enlargement (LAE) (Fisher's exact test, ***p < 0.001).

**Figure 3:**
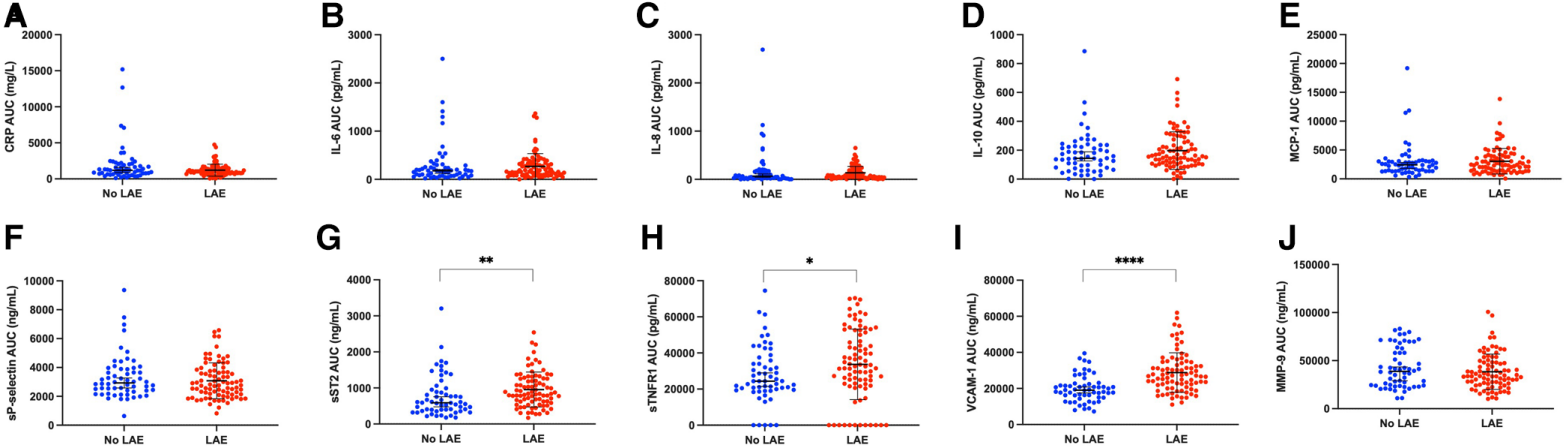
Scatter plot showing levels of circulating inflammatory markers according to the presence of left atrial enlargement (LAE): (**A**,**C**) reactive protein (CRP), (**B**) interleukin-6 (IL-6), (**C**) interleukin-8 (IL-8), (**D**) interleukin-10 (IL-10), (**E**) monocyte chemoattractant protein-1 (MCP-1), (**F**) soluble P-selectin (sP-selectin), (**G**) soluble form suppression of tumorigenicity 2 (sST2), (**H**) soluble tumor necrosis factor receptor I (sTNFR1), (**I**) vascular cellular adhesion molecule-1 (VCAM-1), (**J**) matrix metalloproteinase-9 [Whitney test, *p < 0.05, **p < 0.01, ****p < 0.0001, error bar (mean ± standard deviation) in black].

**Figure 4:**
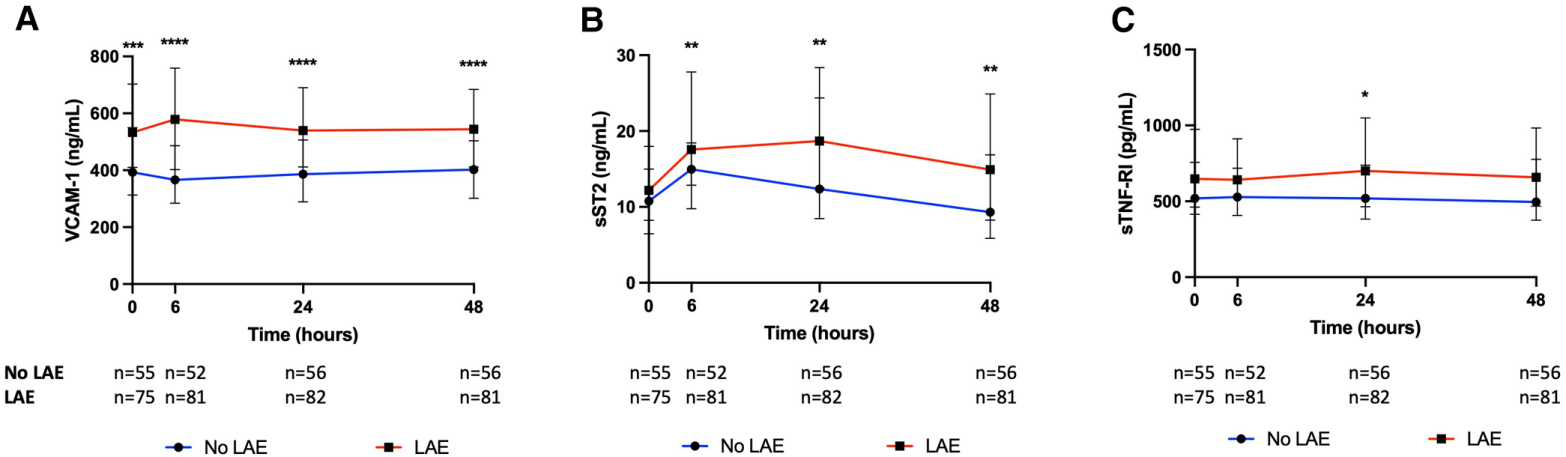
Kinetics of (**A**) vascular cellular adhesion molecule-1 (VCAM-1), (**B**) soluble form suppression of tumorigenicity 2 (sST2), and (**C**) soluble tumor necrosis factor receptor I (sTNFR1) according to the presence of left atrial enlargement (LAE) (Mann–Whitney test, *p < 0.05, **p < 0.01, ***p < 0.001, ****p < 0.0001).

On univariable analysis, patients with LAE were older, were mostly female, had a higher NIHSS score and blood glucose level at admission, had more frequent hypertension, and a cardioembolic source of AIS, such as AF, while they were less frequently current smokers and had a lower rate of tandem occlusion than patients without LAE (Figure 2). Patients with LAE had higher VCAM-1, sST2, and sTNFR1 AUC than patients without LAE (Table 2, Figure 3). VCAM-1 levels differed from admission and remained higher within the first 48 h in patients with LAE, whereas sST2 and sTNFR1 levels differed at 6 h and at 24 h, respectively (Figure 4). Following multivariable analysis, among circulating markers, only high VCAM-1 and sST2 AUC were associated with LAE, together with AF, in both models (Table 3).

**Table 2:** Circulating inflammatory marker levels according to the presence of left atrial enlargement (LAE).

AUC	No LAE (*n* = 58)	LAE (*n* = 85)	*p*-value
CRP (mg/L)	1,189 [711–2,072]	1,037 [736–1,436]	0.23
IL-6 (pg/ml)	176.1 [82.1–288.1]	189.8 [93.2–341.3]	0.38
IL-8 (pg/ml)	67.5 [27.3–176.8]	80.6 [41.3–201.4]	0.35
IL-10 (pg/ml)	143.9 [79.1–227.6]	158.2 [107.5–261.0]	0.21
MCP-1 (pg/ml)	2,418 [1,340–3,063]	2,475 [1,467–3,878]	0.34
sP-selectin (ng/ml)	2,929 [2,395–3,894]	2,817 [2,145–3,751]	0.36
sST2 (ng/ml)	587.7 [396.0–952.4]	840.1 [623.2–1,258.1]	**0**.**002**
sTNFR1 (pg/ml)	24,392 [19,581–35,830]	32,549 [22,980–50,060]	**0**.**01**
VCAM-1 (ng/ml)	19,017 [15,031–22,409]	27,087 [21,918–34,037]	**<0**.**0001**
MMP-9 (ng/ml)	38,866 [24,210–62,675]	33,954 [26,271–48,071]	0.27

LAE, left atrial enlargement; AUC, area under the curve; CRP, C-reactive protein; IL-6, interleukin-6; IL-8, interleukin-8; IL-10, interleukin-10; MCP-1, monocyte chemoattractant protein-1; sP-selectin, soluble P-selectin; sST2, soluble form suppression of tumorigenicity 2; sTNFR1, soluble tumor necrosis factor receptor I; VCAM-1, vascular cellular adhesion molecule-1; MMP-9, matrix metalloproteinase-9.

The bold values indicate a *p*-value < 0.05.

**Table 3:** Factors associated with left atrial enlargement (LAE).

	Odds ratio (95% CI)	*p*-value
Multivariable analysis including VCAM-1 AUC		
High VCAM-1 AUC	9.13 (3.21–25.95)	**<0**.**001**
Age^a^	1.21 (0.83–1.77)	0.31
Male	0.52 (0.19–1.42)	0.20
Hypertension	2.43 (0.85–6.95)	0.10
NIHSS score	1.06 (0.97–1.15)	0.20
Glucose level at admission^b^	1.18 (0.84–1.66)	0.34
Atrial fibrillation	7.47 (2.37–23.60)	**0**.**001**
Tandem occlusion	0.38 (0.11–1.28)	0.12
Multivariable analysis including sST2 AUC		
High sST2 AUC	3.40 (1.68–6.86)	**0**.**001**
Age^a^	1.90 (1.46–2.48)	**<0**.**001**
Male	0.45 (0.22–0.92)	**0**.**03**
Hypertension	2.46 (1.24–4.92)	**0**.**01**
Current smoking	0.31 (0.14–0.71)	**0**.**01**
Glucose level at admission^b^	1.37 (1.07–1.75)	**0**.**01**
Atrial fibrillation	14.07 (5.67–34.90)	**<0**.**001**
Tandem occlusion	0.44 (0.20–0.97)	**0**.**04**

CI, confidence interval; VCAM-1, vascular cellular adhesion molecule-1; AUC, area under the curve; sST2, soluble form suppression of tumorigenicity 2.

Multivariable model including VCAM-1 AUC along with age, sex, hypertension, NIHSS score, glucose level on admission, atrial fibrillation, and tandem occlusion (current smoking not retained by the backward selection).

Multivariable model including sST2 AUC along with age, sex, hypertension, current smoking, glucose level on admission, atrial fibrillation, and tandem occlusion (NIHSS score not retained by the backward selection).

The bold values indicates a *p* value  < 0.05.

^a^ Per 10-year increase.

^b^ Per a 1-mmol/L increase.

## 4. Discussion

**Figure 5:**
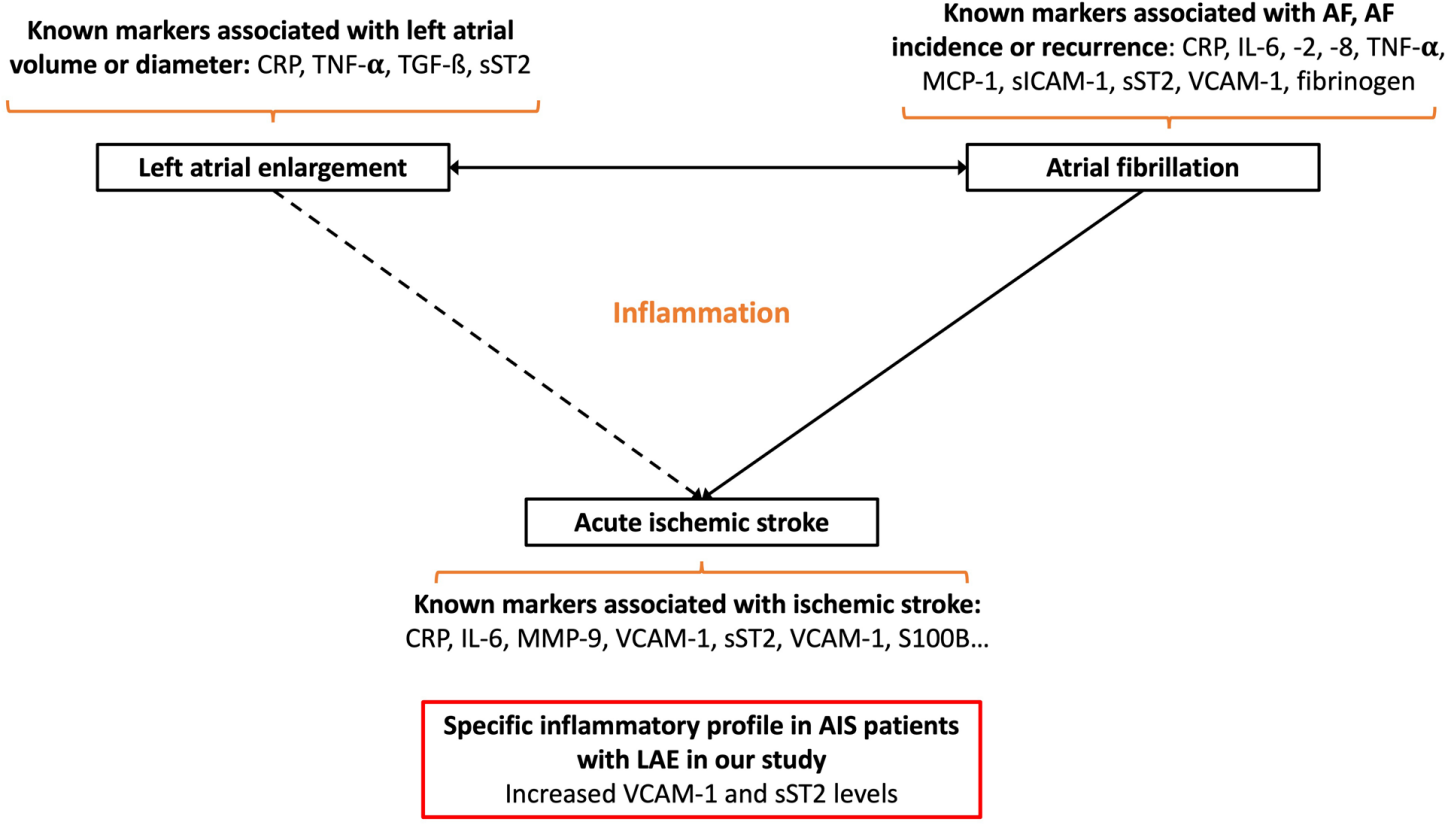
Schematic summarizing the main link between atrial fibrillation, left atrial enlargement, and acute ischemic stroke: known circulating inflammatory markers associated with these conditions and the main results of our study (CRP, C-reactive protein; sICAM-1, soluble intercellular adhesion molecule-1; IL-6, interleukin-6; IL-8, interleukin-8; IL-10, interleukin-10; MMP-9, matrix metalloproteinase-9; MCP-1, monocyte chemoattractant protein-1; S100B, S100 calcium binding protein B; sP-selectin, soluble P-selectin; sST2, soluble form suppression of tumorigenicity 2; sTNFR1, soluble tumor necrosis factor receptor I; TGF-β, transforming growth factor-β; TNF-α, tumor necrosis factor-α; VCAM-1, vascular cellular adhesion molecule-1).

Several studies have shown that the level of systemic markers of inflammation correlated with left atrial volume. However, the impact of the inflammatory substrate underlying atrial myopathy on the overall inflammatory response promoted by AIS remains poorly documented. We demonstrated that VCAM-1 and sST2 levels were higher within the first 48 in AIS patients with LAE (Figure 5).

Higher baseline and persistently increased VCAM-1 levels in patients with LAE argue in favor of a chronic condition linked to LAE. VCAM-1 is involved in post-ischemic neuroinflammation as it permits extravasation of leukocytes through the vascular wall into brain tissue (24). Both systemic and intracranial levels of VCAM-1 during MT are associated with increased infarct and edema volumes and a worsened prognosis (25). Previous studies have also reported a link between VCAM-1 expression and AF. Two population-based cohort studies have shown an association between VCAM-1 levels and AF incidence (13, 14). In addition, patients with AF had higher VCAM-1 levels than those in sinus rhythm (26). Experimental studies have shown that rapid atrial pacing increases endocardial VCAM-1 expression, demonstrating that AF itself increases VCAM-1 expression (27).

With regard to sST2, higher levels from 6 h after admission in patients with LAE may suggest a differential postischemic inflammatory response. sST2 acts as a decoy receptor of IL-33 and therefore inhibits the M2 polarization effect of IL-33/ST2 signaling on microglial cells and macrophages (28). sST2 is associated with an expansion of the lesion within penumbra in AIS patients treated with MT and also with functional outcome and death (19, 29, 30). sST2 levels, which have proven their positive correlation with the left ventricular diameter and ejection fraction, and their diagnostic and prognostic value in heart failure, are also higher in patients with AF and are correlated with the left atrial diameter (15, 31). Our results would be helpful in further studies when interpreting the circulating levels of inflammatory markers in AIS patients.

Apart from circulating inflammatory markers, multivariable analysis demonstrated an association between LAE and higher age, female sex, hypertension, lack of current smoking, atrial fibrillation, higher glucose level on admission, and lack of tandem occlusion. These results are in line with previous studies conducted on non-stroke patients except for no current smoking and tandem occlusion (32–35). The latter two factors are more likely associated with large-artery atherosclerosis than with AF, which may explain why they are less likely associated with LAE in this specific population of AIS patients.

The strength of our study lies in its originality and the quality of the data based on a well-characterized and homogeneous cohort, which benefited from a sequential assessment of circulating inflammatory markers and MRI. Our study has some limitations, such as sample size, a monocentric design, and a narrow panel of markers. Indeed, other markers of endothelial dysfunction, such as von Willebrand's factor and soluble thrombomodulin, which correlate with left atrial volume in patients with lone permanent non-rheumatic AF, might have been of greater interest (36).

## 5. Summary/conclusion

These results call for a more differentiated analysis of the inflammatory response in AIS depending on the presence of atrial myopathy. Future studies should clarify whether this specific inflammatory profile affects the severity and ischemic recurrence in AIS patients with LAE.

## Data Availability

The raw data supporting the conclusions of this article will be made available by the authors without undue reservation.
